# PFKFB4 promotes angiogenesis via IL-6/STAT5A/P-STAT5 signaling in breast cancer

**DOI:** 10.7150/jca.66773

**Published:** 2022-01-01

**Authors:** Dan Li, Jiaping Tang, Ruifang Gao, Jinxin Lan, Wenzhi Shen, Yanhua Liu, Yanan Chen, Hongwei Sun, Jie Yan, Yongwei Nie, Na Luo

**Affiliations:** 1 Department of Anatomy and Histology, School of Medicine, Nankai University, Tianjin 300071, China.; 2 Tianjin Institute of Medical & Pharmaceutical Sciences, Tianjin 300131, China.; 3 Department of Pathology and Institute of Precision Medicine, Jining Medical University, Jining 272067, China.; 4 Tianjin Key Laboratory of Tumour Microenvironment and Neurovascular Regulation, Nankai University, Tianjin 300071, China.; 5 Experimental Center of Operations, Chinese People's Armed Police Force Command College, Tianjin 300250, China.

**Keywords:** PFKFB4, angiogenesis, IL-6/STAT5A/P-STAT5, breast cancer

## Abstract

Breast cancer has become the most newly-diagnosed cancer and the 5^th^ leading cause of cancer death worldwide. The 5-year survival rate of breast cancer is about 90%. However, the 5-year survival rate drops to <30% when metastasis to distant sites occurs. The blood vessel formation (i.e., angiogenesis) plays a crucial role during the metastatic process. In this study, we investigated the role of PFKFB4 in angiogenesis of breast cancer. Employing *in vitro* HUVEC tube formation or *in vivo* orthotopic mouse model, and gene editing or specific small inhibitors strategy, and utilizing qPCR, western blot, ELISA, or immunofluorescent/immunohistochemistry staining methods, we found the following: 1) PFKFB4 upregulates IL-6 expression via NF-κB signaling in breast cancer cells; 2) PFKFB4-induced lactate secretion contributes to NF-κB activation in breast cancer cells; 3) IL-6 elicits angiogenesis via STAT5A/P-STAT5 in HUVEC; 4) 5-MPN (a specific PFKFB4 inhibitor) suppresses angiogenesis *in vitro* and *in vivo*. Our findings suggest a potential strategy whereby 5-MPN may lead to an improved therapeutic outcome for breast cancer patients.

## Introduction

Breast cancer has become the most newly-diagnosed cancer and the 5^th^ leading cause of cancer death worldwide, according to global cancer statistics 2020 1. The 5-year survival rate of breast cancer is about 90%. However, the 5-year survival rate drops to <30% when metastasis to distant sites occurs 2. During this metastatic process, the blood vessel formation (i.e., angiogenesis) plays a crucial role 3.

Tumors utilize passive transport for gas exchange and nutrient delivery during the early stages of tumor development. However, tumors develop their own blood supply due to lack of oxygen provision and accumulation of metabolism waste during the later stages as the tumor grows in size 4. Under such hypoxic and acidic conditions, hypoxia inducible factors (HIFs) are activated along with the later induction of vascular endothelial growth factor (VEGF), platelet derived growth factor (PDGF), and epidermal growth factor (EGF) to promote angiogenesis 5. Angiogenesis describes the formation of new blood vessels from pre-existing blood vessels. Tumor angiogenesis occurs in a highly disorganized, tortuous, and misshapen pattern compared with normal physiological angiogenesis 6. This pattern of tumor angiogenesis leads to blood flow disturbances and eventually to permanent or temporary hypoxia. Moreover, tumor angiogenesis results in impaired vascular perfusion of the tumor which hampers the delivery of anti-tumor agents and mitigates the efficacy of tumor treatments 6.

Hypoxia induces the expression of 6-phosphofructo-2-kinase/fructose-2, 6-bisphosphatase 4 (PFKFB4) which is essential for cancer progression 7, 8. PFKFB4 belongs to the PFKFB family with bifunctional kinase/phosphatases activity that regulates the concentration of the glycolytic byproduct fructose-2,6-biphosphate (F2,6BP). F2,6BP is the allosteric agonist of phosphofructokinase-1 (PFK-1) which is the rate-limiting enzyme in glycolysis. PFK-1 irreversibly catalyzes the synthesis of fructose-1,6-bisphosphate (F1,6BP) from fructose-6-phosphate (F6P) thereby ensuring that glycolysis proceeds^9^. In addition, PFKFB4 activates the steroid receptor coactivator-3 (SRC-3) which regulates activity of multiple transcription factors 10. This suggests that PFKFB4 plays a pivotal role not only in the regulation of glycolysis but also of transcription. Furthermore, PFKFB4 participates in multiple steps of cancer development, such as 1) PFKFB4 mediates the tumorigenesis of thyroid cancer by negatively regulating expression of histone acetyltransferase GCN5 11, 2)PFKFB4 promotes breast cancer metastasis via induction of hyaluronan production 12, and 3) PFKFB4 modulates chemoresistance of small-cell lung cancer by regulating autophagy 13.

In this study, we investigated the role of PFKFB4 in angiogenesis of breast cancer. We found the following: 1) PFKFB4 upregulates IL-6 expression via NF-κB signaling in breast cancer cells; 2) PFKFB4-induced lactate secretion contributes to NF-κB activation in breast cancer cells; 3) IL-6 elicits angiogenesis via STAT5A/P-STAT5 in HUVEC; 4) 5-MPN (a specific inhibitor of PFKFB4) suppresses angiogenesis *in vitro* and *in vivo*. Our findings suggest a potential strategy whereby 5-MPN either as a single agent or in combination with currently available treatments may lead to an improved therapeutic outcome for breast cancer patients.

## Materials and Methods

### Cells and Treatment

The human breast carcinoma cells MDA-MB-231, T47D and human umbilical venin endothelial cells (HUVEC) were obtained from Core Technology Facility of Center for Excellence in Molecular Cell Science, CAS. The MDA-MB-231 and T47D cells were cultured in high glucose Dulbecco's modified Eagle's medium (06-1055-57-1ACS, DMEM, Biological Industries, Israel) containing 10% fetal bovine serum (04-001-1ACS, Biological Industries, Israel) and 100U/ml penicillin/streptomycin (SV30010, Hyclone, Logan, UT). The HUVEC were cultured in RPMI 1640 medium (01-100-1ACS, Biological Industries, Israel) containing 10% fetal bovine serum (04-001-1ACS, Biological Industries, Israel) and 100 U/ml penicillin/streptomycin (SV30010, Hyclone, Logan, UT). Cells were cultured at 37°C in a humidified atmosphere with 5% CO_2_.

The MDA-MB-231 and T47D cells were treated either with 10 μM PDTC (S3633, Selleck), 8nM AZD3965 (S7339, Selleck), or with 5 μM 5-MPN (S656801, Sigma-Aldrich) 24hrs for Western blot.

The HUVEC in 6-well plates were either transfected with a siRNA targeting STAT5A (s13535, Ambion), STAT5B (s13539, Ambion) or non-silencing control using Dharmafect1 (T-2001-03, Dharmacon) transfection reagent according to the manufacturer's protocol, or treated with anti-IL-6 (1:1000, WL02841, Wanleibio) 24hrs for Western blot.

### Vector construction and establishment of stable cell lines

For gene overexpression, DNA sequences encoding human PFKFB4 were PCR-amplified from MDA-MB-231 cDNA and cloned into the pLV-EF1α-MCS-IRES-Bsd plasmid (CDNA-pLV03, Biosettia, San Diego, CA, USA). For gene silencing, short hairpin RNAs (shRNAs) targeting human PFKFB4 were cloned into the pLV-H1-EF1α-puro plasmid (SORT-B19, Biosettia, San Diego, CA, USA). The lentiviruses carrying the overexpression vectors, gene silencing vectors or empty vectors were produced according to manufacturer's instruction. Lentivirus-containing medium was applied to MDA-MB-231 and T47D cells in the presence of 8 μg/ml polybrene for 48 hrs, prior to selection with 10 μg/ml blasticidin or 1 μg/ml puromycin for a week to establish stable cell lines. The primers and shRNAs were listed in Supplementary Table 1.

### Endothelial tube formation assay

Wells of 48-well plates were coated with growth factor reduced matrigel (356231, BD Biosciences, Mississauga, ON, Canada). Once polymerized, 4X10^4^ HUVECs were plated per well. Conditioned medium of MDA-MB-231 and T47D cells was added directly to HUVECs for tube formation. After 6hrs, the endothelial cell tube formation were examined and photographed. Tube formation was quantified by counting the number of connected cells in 5 randomly selected fields.

### Isolation of nuclear and cytoplasmic compartments

The nuclear and cytoplasmic compartment proteins of MDA-MB-231 and T47D cells were isolated using a Nuclear and Cytoplasmic Protein Extraction Kit (KGP1100, KeyGEN Biotech, Jiangsu, China) according to the manufacturer's instruction.

### Western blot

Detection of protein expression by Western blot was carried out according to the established protocols described previously 14. Briefly, cells were washed in cold phosphate-buffered saline, collected and lysed in 1X RIPA buffer (50 mM Tris (pH 7.4), 1% NP-40, 150 mM NaCl, 1 mM EDTA, 0.1% SDS, 0.25% sodium deoxycholate, 5 mM NaF, 5mM Na3VO4, 10% glycerol, 1M phenylmethyl-sulphonylfluoride and protease inhibitors) for 30min on ice. Lysates was centrifuged at 13,000 rpm for 15 min at 4 °C. Protein concentrations of the lysates were determined by BCA assay (23225, Thermo Fisher). Samples were separated by 8% SDS-PAGE and transferred to nitrocellulose membrane. Membranes were blocked with 5% non-fat dry milk in tris-buffered saline with 0.1% Tween-20 for 1 hrs at room temperature and then incubated overnight at 4 °C with the appropriate antibody as indicated. Following incubation with appropriate horseradish peroxidase-conjugated secondary antibodies, proteins were visualized using an enhanced chemiluminescence detection system (32106, Thermo Fisher). Western blot was performed using the following antibodies: PFKFB4 (1:1000, ab137785, Abcam), IL-6 (1:1000, WL02841, Wanleibio), IκBα (phosphor S36) (1:1000, ab133462, Abcam), IκBα (1:1000, ab76429, Abcam), p-NF-κBp65 ser536 (1:1000, WL02169, Wanleibio), NF-κBp65 (1:1000, sc-398442, Santa Cruz), β-actin (1:2000, sc-47778, Santa Cruz), Lamin A/C (1:1000, 2032, CST), α-Tubulin (1:1000, 2144, CST), CD31 (1:1000, WL03674, Wanleibio), IL-6R (1:1000, WL02737, Wanleibio), p-STAT1 (Y701) (1:1000, 7649, CST), p-STAT3 (Y705) (1:1000, 9145, CST), p-STAT5 (Y694) (1:1000, 9359, CST), STAT3 (1:1000, 9139, CST) , STAT1 (1:1000, sc-592, Santa Cruz), STAT5A (1:1000, sc-1081, Santa Cruz), STAT5B (1:1000, sc-1656, Santa Cruz).

### Immunofluorescence

The MDA-MB-231 and T47D breast cancer cells were grown on coverslips until 60%-80% confluence. After fixation in 4% paraformaldehyde and permeablization with 0.1% TritonX-100, the cells were incubated with anti-NF-κBp65 (1:200, sc-398442, Santa Cruz), anti-IL-6R (1:200, WL02169, Wanleibio), anti-PFKFB4 (1:200, ab137785, Abcam) antibodies at 4°C overnight. After incubation with Alexa Fluor 488 goat anti-rabbit IgG (1:1000, A32731, Invitrogen, Carlsbad, CA) at room temperature for 1hr, the cells stained with DAPI for nuclear visualization. The immunofluorescent signal was photomicrographs with an Olympus FV1000-IX81 confocal microscope (Olympus, Tokyo, Japan).

### Quantitative RT-PCR

Total RNA of each sample was collected using TRIzol reagent (15596026, Invitrogen), and for first-strand complementary DNA synthesis was performed using M-MLV Reverse Transcriptase (2641A, Takara, Tokyo, Japan). The specific products of IL-6, VEGFA, PDGFA, bFGF, IL-8, MCT1, MCT3, MCT4 were amplified using a TransStart Green Q-PCR SuperMix kit (AQ101-02, TransGen, Beijing, China). GAPDH was used as a normalization control. The qPCR was performed in a CFXTM Real-Time Thermal cycler (Bio-Rad, Hercules, CA, USA). The primers used were listed in Supplementary Table 2.

### Enzyme-linked immunosorbent assay (ELISA)

Soluble IL-6 in MDA-MB-231 and T47D cell culture supernatant was quantified using the IL-6 (Human) ELISA kit (EH2IL6, Thermo Fisher) according to the manufacturer's protocol. Briefly, the supernatant was added in duplicate to the microplate wells coated with Biotinylated IL-6 antibody, followed with Stripavidin-HRP and TMB substrate incubation. After adding stop solution, the absorbance was measured at 450 nm using a microplate reader (Promega).

### Animal experiments with 5-MPN

PFKFB4-expression or control MDA-MB-231 cells (1 × 10^6^ in 100 μl basic DMEM) were injected into the second mammary fat pad of 8-week-old female NOD/ SCID mice. Following the establishment of tumors (~100mm^3^), the PFKFB4-expression or control MDA-MB-231 mice were randomized to treatment with DMSO or 5-MPN (120 mg/kg, every two days by oral gavage for 10 days). For tumor growth analysis, tumors were measured 2 times weekly with calipers and volume was calculated in mm^3^ using the formula (length x width x width/2). Mice were humanely euthanized after 10-day gavage. Five mice were used for each treatment arm for tumor growth studies. Animal use complied with Nankai University Animal Welfare Guidelines.

### Immunohistochemistry

Paraffin-embedded sections of mouse MDA-MB-231 tissues were deparaffinized and antigen retrieval performed using citrate buffer (pH=6) or Tris EDTA buffer (pH=9), at a temperature of 97 °C for 20 min. After exhaustion of endogenous peroxidase with methanol and hydrogen peroxide, slides were blocked with 0.3% BSA in 0.1 mol/L of tris-buffered saline for 30 min at room temperature, and incubated with primary antibody against IL-6 (1:200, WL02841, Wanleibio), p-NF-κBp65 ser536 (1:200, WL02169, Wanleibio), IL-6R (1:200, WL02169, Wanleibio), CD31 (1:200, WL03674, Wanleibio), STAT5A (1:200, sc-1081, Santa Cruz), p-STAT5 (Y694) (1:200, 9359, CST) overnight at 4 °C. Antibody binding was detected using a peroxidase-conjugated secondary antibody at 37 °C for 30 min. A DAB Substrate Kit was used to perform the chromogenic reaction.

### Statistical analysis

All data were analyzed using GraphPad Prism5 software (GraphPad Software, San Diego, CA, USA). Results were expressed as means ± SD with the exception of animal model data, which were expressed as mean ± SEM. P values were calculated using a two-tailed Student's t-test (two groups) or one-way ANOVA (more than 2 groups) unless otherwise noted. The results were considered statistically significant when *P<0.05, **P<0.01, ***P<0.001.

## Results

### PFKFB4 promotes endothelial cell tube formation via IL-6

**Figure 1:**
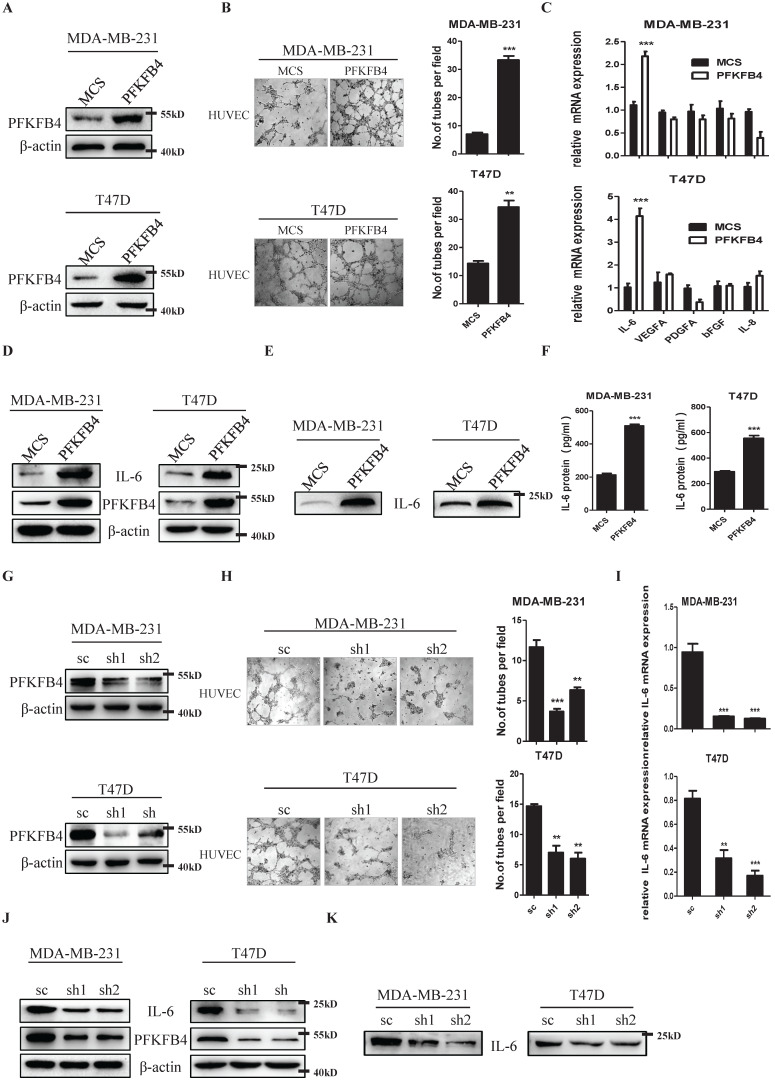
** PFKFB4 promotes endothelial cell tube formation via IL-6. A)** The Western blot analysis showed the establishment of MDA-MB-231/PFKFB4 and T47D/PFKFB4 cell lines. β-actin was used as a loading control. **B)** The representative photomicrographs of 3 independent tube formation assays showed that ectopic expression of PFKFB4 in MDA-MB-231 and T47D cells promoted tube formation compared to the MCS group. The bar graph shows the quantification of tube formation assay that illustrates ectopic expression of PFKFB4 in MDA-MB-231 and T47D cells significantly increased tube formation versus the MCS group (7.00±0.58 versus 33.33±1.45; 14.33±0.88 versus 24.33±2.33, respectively; ** p<0.01, *** p<0.001). **C)** The quantitative PCR showed that ectopic expression of PFKFB4 in MDA-MB-231 and T47D cells significantly increased relative expression of IL-6 (1.11±0.07 versus 2.18±0.10; 1.03±0.17 versus 4.14±0.34, respectively; *** p<0.001), but not VEGFA, PDGFA, bFGF, or IL-8. **D)** The Western blot analysis showed ectopic expression of PFKFB4 in MDA-MB-231 and T47D cells increased IL-6 expression. β-actin was used as a loading control. **E)** The Western blot analysis showed ectopic expression of PFKFB4 in MDA-MB-231 and T47D cells increased IL-6 expression in the conditioned medium. **F)** The ELISA analysis showed that ectopic expression of PFKFB4 in MDA-MB-231 and T47D cells significantly increased IL-6 amount in the conditioned medium versus the MCS group (213.3±8.82 versus 509.3±9.24; 293.7±7.31 versus 554.0±22.30, respectively; *** p<0.001). **G)** The Western blot analysis showed the establishment of MDA-MB-231/shPFKFB4 and T47D/shPFKFB4 cell lines. β-actin was used as a loading control. **H)** The representative photomicrographs of 3 independent tube formation assays showed that knocking down of PFKFB4 in MDA-MB-231 and T47D cells inhibited tube formation compared to the sc group. The bar graph shows the quantification of tube formation assay that illustrates knocking down of PFKFB4 in MDA-MB-231 and T47D cells significantly decreased tube formation versus the sc group (11.67±0.88 versus 3.67±0.33, 6.33±0.33; 14.67±0.33 versus 7.00±1.16, 6.00±1.00, respectively; ** p<0.01, *** p<0.001). **I)** The quantitative PCR showed that knocking down of PFKFB4 in MDA-MB-231 and T47D cells significantly decreased relative expression of IL-6 (0.94±0.10 versus 0.15±0.04, 0.13±0.05; 0.81±0.07 versus 0.32±0.07, 0.17±0.04, respectively; ** p<0.01, *** p<0.001). **J)** The Western blot analysis showed knocking down of PFKFB4 in MDA-MB-231 and T47D cells decreased IL-6 expression. β-actin was used as a loading control. **K)** The Western blot analysis showed knocking down of PFKFB4 in MDA-MB-231 and T47D cells decreased IL-6 expression in the conditioned medium.

We ectopically expressed PFKFB4 in human breast carcinoma MDA-MB-231 and T47D cells and established stable cell lines (i.e., MDA-MB-231/PFKFB4 and T47D/PFKFB4, respectively) (Fig. 1A). The endothelial cell tube formation assay showed that the conditioned medium collected from MDA-MB-231/PFKFB4 and T47D/PFKFB4 cells significantly promoted HUVEC endothelial cell tube formation (Fig. 1B). We next explored cytokine secretion by MDA-MB-231 and T47D cells that promotes HUVEC tube formation due to PFKFB4 ectopic expression. First, the human angiogenesis array results showed that IL-6 altered most in the conditioned medium of MDA-MB-231/PFKFB4 cells, compared with other cytokines (data not shown). Moreover, quantitative PCR showed the consistent results that PFKFB4 ectopic expression significantly increases IL-6, but not VEGFA, PDGFA, bFGF, or IL-8 mRNA expression in MDA-MB-231 and T47D cells (Fig. 1C). Western blot analysis confirmed that PFKFB4 ectopic expression increases IL-6 expression in MDA-MB-231 and T47D cells (Fig. 1D). Furthermore Western blot and IL-6 ELISA showed that PFKFB4 ectopic expression increases IL-6 levels in the conditioned medium of MDA-MB-231 and T47D cells (Fig. 1E & F). To further confirm the above results, we knocked down PFKFB4 expression in MDA-MB-231 and T47D cells and established stable cell lines (i.e., MDA-MB-231/shPFKFB4 and T47D/shPFKFB4, respectively) (Fig. 1G). The endothelial cell tube formation assay showed that PFKFB4 knockdown in MDA-MB-231 and T47D cells significantly inhibits HUVEC endothelial tube formation (Fig 1H). In addition, PFKFB4 knockdown in MDA-MB-231 and T47D cells significantly decreases IL-6 mRNA and protein expression (Fig. 1I & J). Finally, Western blot analysis showed that PFKFB4 knockdown decreases IL-6 levels in the conditioned medium of MDA-MB-231 and T47D cells (Fig. 1K).

### PFKFB4 increases IL-6 expression via the NF-κB signaling pathway in human breast carcinoma cells

**Figure 2:**
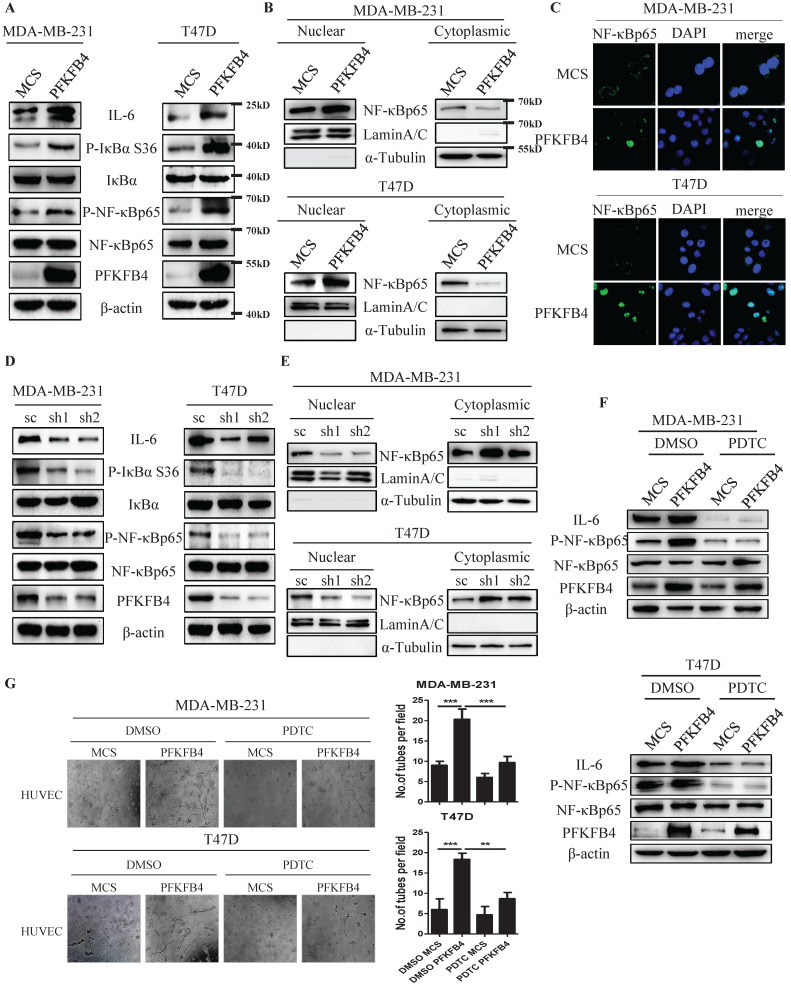
** PFKFB4 increases IL-6 expression via the NF-κB signaling pathway. A)** The Western blot analysis showed that ectopic expression of PFKFB4 in MDA-MB-231 and T47D cells increased IκBα (S36) and NF-κB p65 phosphorylation. IκBα, NF-κB p65, and β-actin were used as a loading control. **B)** The Western blot analysis showed that ectopic expression of PFKFB4 in MDA-MB-231 and T47D cells promoted NF-κB p65 translocation from cytoplasmic to nuclear compartment, and increased NF-κB p65 accumulation in the nuclear compartment. Lamin A/C was used as a loading control of nuclear protein fraction and α-tubulin was used as a loading control of cytoplasmic protein fraction. **C)** The representative immunofluorescent photomicrographs illustrated that ectopic expression of PFKFB4 in MDA-MB-231 and T47D cells promoted NF-κB p65 (green) immunostaining in the nuclear compartment. The nuclei (blue) were stained using DAPI. **D)** The Western blot analysis showed that knocking down of PFKFB4 in MDA-MB-231 and T47D cells decreased IκBα (S36) and NF-κB p65 phosphorylation. IκBα, NF-κB p65, and β-actin were used as a loading control. **E)** The Western blot analysis showed that knocking down of PFKFB4 in MDA-MB-231 and T47D cells decreased NF-κB p65 accumulation in the nuclear compartment. Lamin A/C was used as a loading control of nuclear protein fraction and α-tubulin was used as a loading control of cytoplasmic protein fraction. **F)** The Western blot analysis showed that PDTC (a NF-κB inhibitor) treatment inhibited PFKFB4-induced NF-κB p65 phosphorylation and IL-6 expression in MDA-MB-231 and T47D cells. β-actin was used as a loading control. **G)** The representative photomicrographs of 3 independent tube formation assays showed that PDTC treatment inhibited PFKFB4-induced tube formation compared to the DMSO/PFKFB4 group. The bar graph shows the quantification of tube formation assay that illustrates PDTC treatment significantly decreased PFKFB4-induced tube formation versus the DMSO/PFKFB4 group (20.33±1.45 versus 9.67±0.88; 18.33±0.88 versus 8.67±0.88, respectively; ** p<0.01, *** p<0.001).

Since the IL-6 promoter region has a putative NF-κB binding-site 15, we investigated whether PFKFB4 increases IL-6 expression via NF-κB signaling pathway in MDA-MB-231 and T47D cells. Western blot analysis showed that PFKFB4 ectopic expression increases IκBα phosphorylation which leads to IκBα degradation. A rise in IκBα degradation induces further NF-κB phosphorylation and activation (Fig. 2A). Additionally, Western blot analysis and immunofluorescence staining showed that PFKFB4 ectopic expression facilitates more NF-κB translocation into the nucleus which results in less NF-κB within the cytoplasm (Fig. 2B & C). Our results also indicated that PFKFB4 knockdown decreases IκBα phosphorylation which leads to less NF-κB phosphorylation and less NF-κB translocation into the nucleus (Fig. 2D & E).

To further confirm that PFKFB4 increases IL-6 expression via the NF-κB signaling pathway, we treated MDA-MB-231 and T47D cells with pyrrolidine dithiocarbamate (PDTC), a NF-κB inhibitor. Our results showed that PDTC treatment inhibits PFKFB4-induced NF-κB phosphorylation and downstream IL-6 expression (Fig. 2F). Moreover, the endothelial cell tube formation assay showed that the conditioned medium collected from PDTC-treated MDA-MB-231/PFKFB4 and T47D/PFKFB4 cells inhibits PFKFB4-induced HUVEC tube formation (Fig. 2G).

Previous reports indicate that the mutation of Lys173 to Ala in PFKFB4 results in a reduction of both ATP binding and F6P binding 16. Our previous research found that the PFKFB4^K173A^ mutation plays a key role in PFKFB4-mediated glycolysis and maintenance of breast cancer stemness 17. So, we next investigated whether the PFKFB4^K173A^ mutation also plays a key role in PFKFB4-induced NF-κB signaling pathway activation and IL-6 expression. Western blot analysis showed that the PFKFB4^K173A^ mutation (mut) impedes NF-κB phosphorylation and IL-6 expression compared to PFKFB4 wild type (PFKFB4) (Supp. Fig. 1A). Immunofluorescence staining showed that the PFKFB4^K173A^ mutation inhibits NF-κB translocation into the nucleus compared to PFKFB4 wild type (Supp. Fig. 1B).

### PFKFB4-induced lactate secretion contribute to the initiation of NF-κB signaling in human breast carcinoma cells

**Figure 3:**
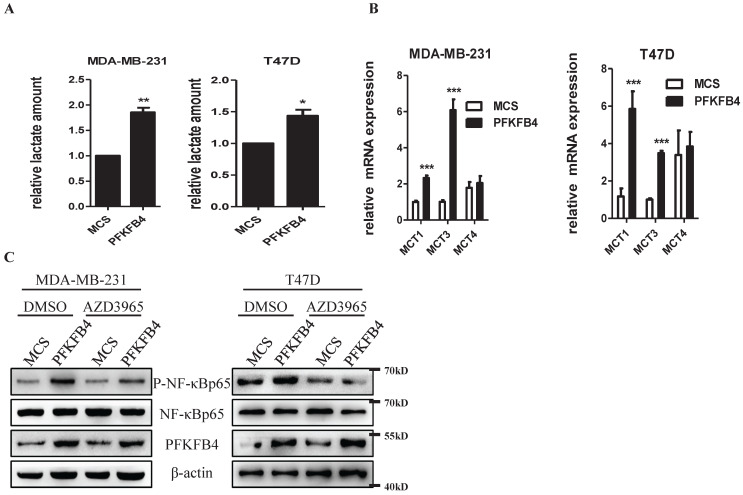
** PFKFB4-induced lactate secretion contributes to the initiation of NF-κB signaling. A)** This bar graph shows the quantification of relative lactate amount that illustrates PFKFB4 significantly increased lactate production and secretion in MDA-MB-231 and T47D cells (1 versus 1.85±0.09; 1 versus 1.44±0.10, respectively; * p<0.05, ** p<0.01). **B)** The quantitative PCR showed that PFKFB4 significantly increased relative expression of MCT1 and MCT3 (MCT1: 1.01±0.06 versus 2.32±0.14; 1.18±0.43 versus 5.85±0.94; MCT3: 1.01±0.86 versus 6.08±0.60; 1.01±0.07 versus 3.48±0.13, respectively; *** p<0.001), but not MCT4 in MDA-MB-231 and T47D cells. **C)** The Western blot analysis showed that AZD3965 (a MCT1 inhibitor) treatment inhibited PFKFB4-induced NF-κB p65 phosphorylation in MDA-MB-231 and T47D cells. β-actin was used as a loading control.

Previous reports have indicated that lactate activates NF-κB signaling pathway in endothelial cells and trabecular meshwork cells 18, 19. Since we have previously reported that PFKFB4 ectopic expression augments lactate secretion in ZR-75-1 and SKBR3 cells 17, we presently studied whether PFKFB4-induced lactate secretion activates NF-κB in MDA-MB-231 and T47D cells. In this regard, first, PFKFB4 ectopic expression also augments lactate secretion in MDA-MB-231 and T47D cells (Fig. 3A). Next, we examined the role of monocarboxylate transporter (MCT; a passive lactate-proton symporter) expression in MDA-MB-231/PFKFB4 and T47D/PFKFB4 cells. Quantitative PCR results showed that MCT1 and MCT3 expression significantly increased in MDA-MB-231/PFKFB4 and T47D/PFKFB4 cells, but MCT4 expression did not (Fig. 3B). Western blot results showed that AZD3965 treatment (a MCT1 inhibitor) partially suppresses PFKFB4-induced NF-κB phosphorylation in MDA-MB-231 and T47D cells (Fig. 3C). In addition, the PFKFB4^K173A^ mutation that impedes NF-κB phosphorylation and IL-6 expression also showed a defect in lactate secretion compared to PFKFB4 wild type (Supp. Fig. 1C). The above-mentioned results indicate that PFKFB4-induced lactate secretion contributes to the initiation of NF-κB signaling in MDA-MB-231 and T47D cells.

### IL-6 induces STAT5A phosphorylation in HUVEC cells

**Figure 4:**
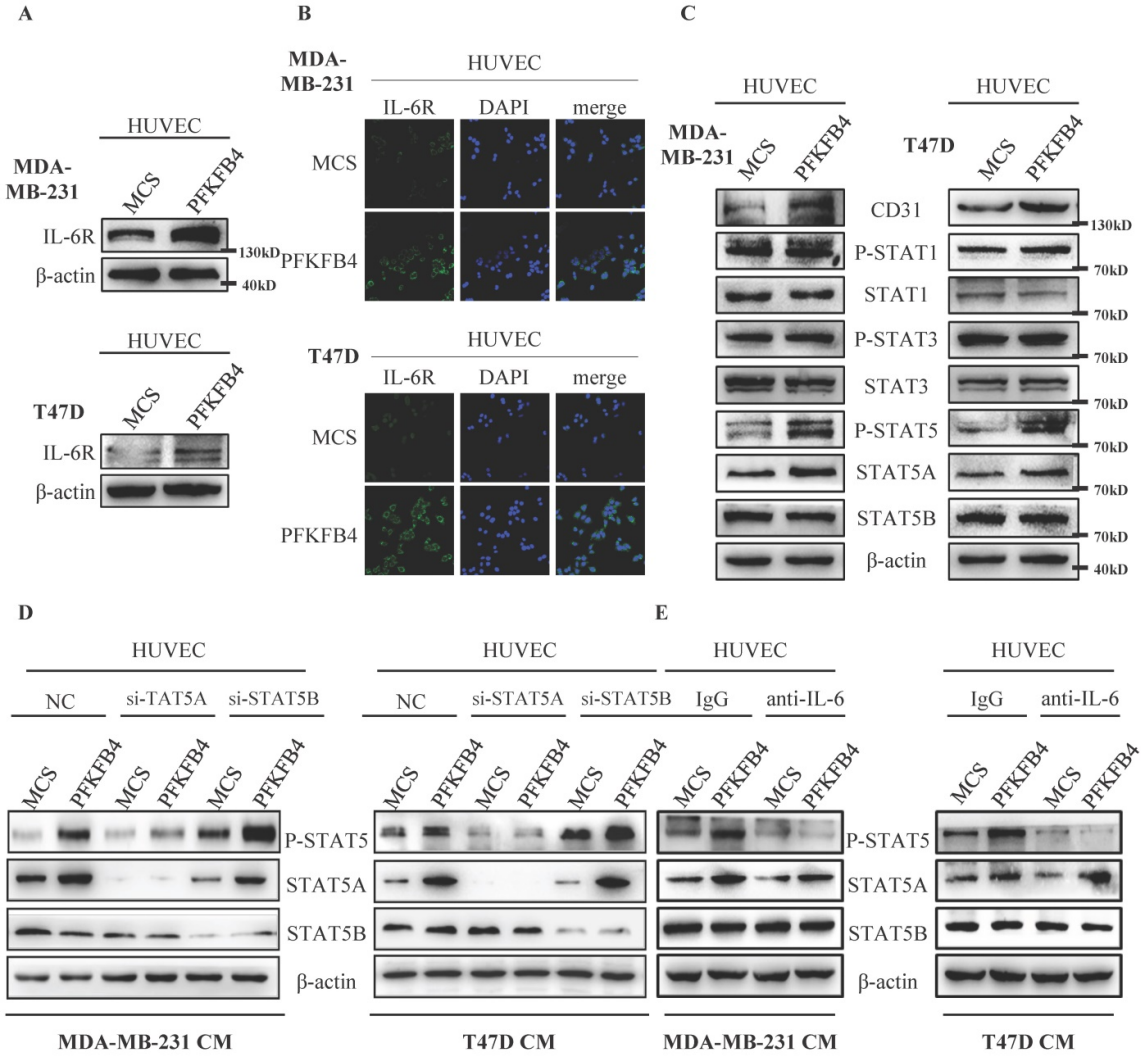
** IL-6 induces STAT5 phosphorylation in HUVEC cells. A)** The Western blot analysis showed that conditioned medium collected from MDA-MB-231/PFKFB4 and T47D/PFKFB4 cells induced IL-6R expression of HUVEC cells. β-actin was used as a loading control. **B)** The representative immunofluorescent photomicrographs illustrated that conditioned medium collected from MDA-MB-231/PFKFB4 (upper panel) and T47D/PFKFB4 (lower panel) augmented IL-6R immunostaining (green) of HUVEC cells. The nuclei (blue) were stained using DAPI. **C)** The Western blot analysis showed that conditioned medium collected from MDA-MB-231/PFKFB4 and T47D/PFKFB4 cells induced STAT5 phosphorylation, STAT5A and CD31 expression of HUVEC cells, but not STAT1 or STAT3 phosphorylation. STAT1, STAT3, STAT5B, and β-actin were used as a loading control. **D)** The Western blot analysis showed that knocking down of STAT5A, but not STAT5B expression in HUVEC, abolished STAT5 phosphorylation promoted by the conditioned medium collected from MDA-MB-231/PFKFB4 and T47D/PFKFB4 cells. β-actin was used as a loading control. E) The Western blot analysis showed that anti-IL-6 treatment of HUVEC blocked STAT5 phosphorylation prompted by the conditioned medium collected from MDA-MB-231/PFKFB4 and T47D/PFKFB4 cells. β-actin was used as a loading control.

We investigated the mechanism by which IL-6 promotes tube formation in HUVEC endothelial cells. Western blot analysis and immunofluorescence staining showed that the conditioned medium collected from MDA-MB-231/PFKFB4 and T47D/PFKFB4 cells increases IL-6R expression in HUVEC endothelial cells which is comparable to PFKFB4-induced IL-6 expression in MDA-MB-231 and T47D cells (Fig. 4A & B). Since IL-6 functions mainly via the JAK/STAT pathway 20-22, we then investigated which JAK/STAT members are involved in IL-6-mediated HUVEC tube formation. Western blot analysis showed that the conditioned medium collected from MDA-MB-231/PFKFB4 or T47D/PFKFB4 cells increases STAT5 phosphorylation (P-STAT5), but not STAT1 phosphorylation (P-STAT) or STAT3 phosphorylation (P-STAT3) in HUVEC cells. Furthermore, we found that the conditioned medium collected from MDA-MB-231/PFKFB4 and T47D/PFKFB4 cells increases STAT5A expression, but not STAT5B expression in HUVEC endothelial cells. Finally, our results indicated that the conditioned medium collected from MDA-MB-231/PFKFB4 or T47D/PFKFB4 cells also increases CD31 (an endothelial cell marker for angiogenesis) expression (Fig. 4C).

In order to investigate which STAT5 member contributes to STAT5 phosphorylation, we knocked down STAT5A or STAT5B expression using the specific siRNA, respectively. Western blot analysis showed that STAT5A knockdown decreases P-STAT5, whereas STAT5B knockdown did not alter P-STAT5 (Fig. 4D). These results indicate that STAT5A (but not STAT5B) plays a role in IL-6-induced P-STAT5 in HUVEC endothelial cells.

Furthermore, to confirm that IL-6 stimulates P-STAT5 in HUVEC endothelial cells, we pre-incubated HUVEC cells with anti-IL-6 antibody or control IgG before treatment with the conditioned medium collected from MDA-MB-231/PFKFB4 and T47D/PFKFB4 cells. Western blot analysis showed that pre-incubation with anti-IL-6 antibody blocks P-STAT5 prompted by the conditioned medium collected from MDA-MB-231/PFKFB4 and T47D/PFKFB4 cells compared to control IgG in HUVEC cells (Fig. 4E). These results indicate that that IL-6 secreted by MDA-MB-231/PFKFB4 and T47D/PFKFB4 cells induces STAT5A/P-STAT5 in HUVEC endothelial cells.

### 5-MPN treatment inhibits PFKFB4-induced angiogenesis signaling *in vitro*

**Figure 5:**
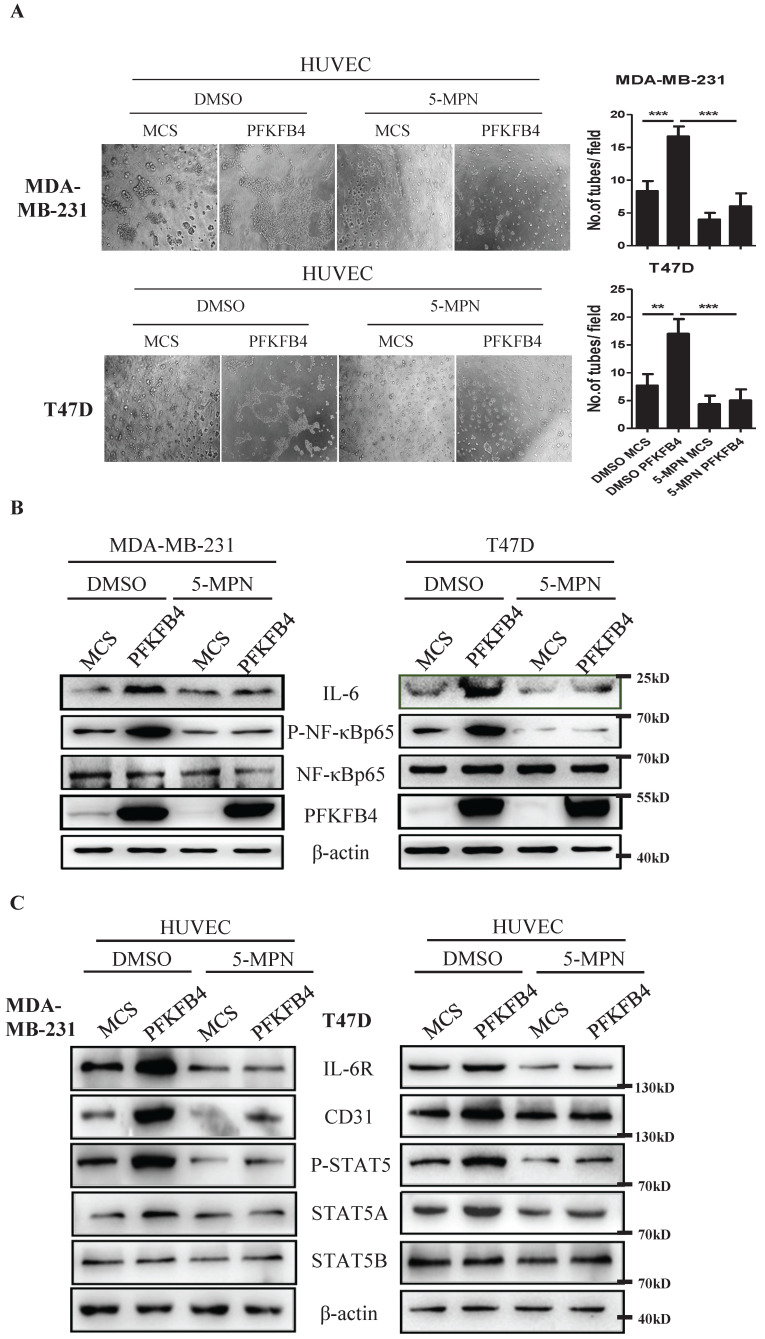
** 5-MPN treatment inhibits PFKFB4-induced angiogenesis signaling *in vitro*. A)** The representative photomicrographs of 3 independent tube formation assays showed that 5-MPN treatment of MDA-MB-231 and T47D cells inhibited PFKFB4-induced HUVEC tube formation compared to the DMSO/PFKFB4 group. This bar graph shows the quantification of tube formation assay that illustrates 5-MPN treatment of MDA-MB-231 and T47D cells significantly decreased HUVEC tube formation versus the DMSO/PFKFB4 group (16.67±0.88 versus 6.00±1.16; 17.00±1.53 versus 5.00±1.16, respectively; ** p<0.01, *** p<0.001). **B)** The Western blot analysis showed that 5-MPN treatment of MDA-MB-231 and T47D cells diminished PFKFB4-induced NF-κB phosphorylation and IL-6 expression. β-actin was used as a loading control. **C)** The Western blot analysis showed that 5-MPN treatment of MDA-MB-231 and T47D cells diminished PFKFB4-induced STAT5 phosphorylation and STAT5A, IL-6R, CD31 expression in HUVEC cells. β-actin was used as a loading control.

Our earlier findings showed that 5-MPN (a specific PFKFB4 inhibitor) suppresses CD44ICD/PFKFB4-induced tumor development 17. Therefore, we explored whether 5-MPN would inhibit PFKFB4-induced angiogenesis signaling. The endothelial cell tube formation assay showed that 5-MPN treatment suppresses PFKFB4-induced HUVEC tube formation compared to the DMSO/PFKFB4 group in MDA-MB-231 and T47D cells (Fig. 5A). Western blot analysis showed that 5-MPN treatment inhibits PFKFB4-induced NF-κB phosphorylation and IL-6 expression compared to the DMSO/PFKFB4 group in MDA-MB-231 and T47D cells (Fig. 5B). Comply with this, 5-MPN treatment hinders PFKFB4-induced lactate secretion (Supp. Fig. 1D). In addition, we found that 5-MPN treatment inhibits the MDA-MB-231/PFKFB4 and T47D/PFKFB4 conditioned medium-induced IL-6R, STAT5A/P-STAT5 and CD31 expression in HUVEC endothelial cells compared to DMSO/PFKFB4 group. However, neither 5-MPN treatment nor PFKFB4 ectopic expression alters STAT5B expression (Fig. 5C). The above results indicate that 5-MPN treatment inhibits PFKFB4-induced angiogenesis signaling.

### 5-MPN treatment inhibits PFKFB4-induced angiogenesis *in vivo*

**Figure 6:**
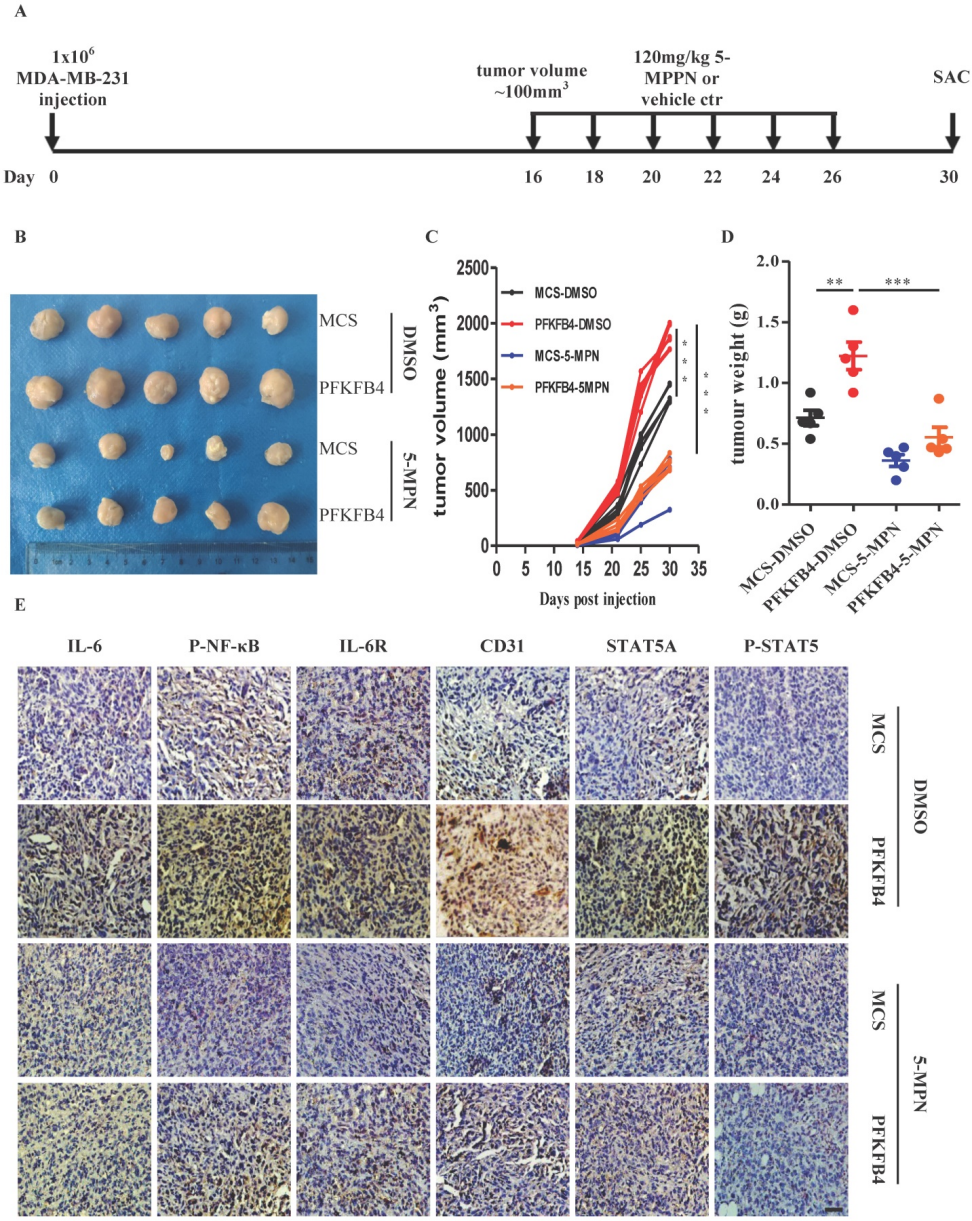
** 5-MPN treatment inhibits PFKFB4-induced angiogenesis *in vivo*. A)** Scheme for the *in vivo* experiment with results shown in panels B, C, D and E. **B)** Photographs of xenograft MDA-MB-231 tumors formed in NOD/SCID mice harvested on Day 30. The photographs qualitatively indicate that ectopic expression of PFKFB4 increased tumor size versus the MCS group, whereas, 5-MPN treatment inhibited PFKFB4-induced growth of tumor size. **C)** Growth curve of xenograft MDA-MB-231 tumors formed in NOD/SCID mice. This graph demonstrates that ectopic expression of PFKFB4 significantly increased tumor size versus the MCS group (1364±36.05 versus 1897+44.31, respectively; *** p<0.001), whereas, 5-MPN treatment significantly inhibited PFKFB4-induced growth of tumor size (1897±44.31 versus 746.6±27.59, respectively; *** p<0.001). Tumor volume = length x width x width/2. **D)** The tumor weight of xenograft MDA-MB-231 tumors formed in NOD/SCID mice. The dot plot demonstrates that ectopic expression of PFKFB4 significantly increased tumor weight versus the MCS group (0.71±0.06 versus 1.22±0.11, respectively; **p<0.01), whereas, 5-MPN treatment significantly inhibited PFKFB4-induced growth of tumor size (1.22±0.11 versus 0.55±0.08, respectively; ***p<0.001). **E)** The representative micrographs of IL-6, P-NF-κB, IL-6R, CD31, STAT5A, and P-STAT5 immunocytochemical staining in xenograft MDA-MB-231 tumors. Ectopic expression of PFKFB4 increased immunocytochemical staining of above-mentioned molecules versus the MCS group, whereas, 5-MPN treatment inhibited PFKFB4-induced immunocytochemical staining of above-mentioned molecules. Scale bar= 50 µm.

In addition to the above-mentioned *in vitro* results concerning 5-MPN treatment, we also found that 5-MPN treatment *in vivo* decreases tumor volume and tumor weight compared with the DMSO group in MDA-MB-231 xenograft tumors (Fig. 6A-D). Furthermore, immunohistochemistry staining showed that 5-MPN treatment decreases PFKFB4-mediated IL-6, p-NF-κB, IL-6R, STAT5A, p-STAT5, and CD31 expression in MDA-MB-231 xenograft tumors (Fig. 6E).

## Discussion

**Figure 7:**
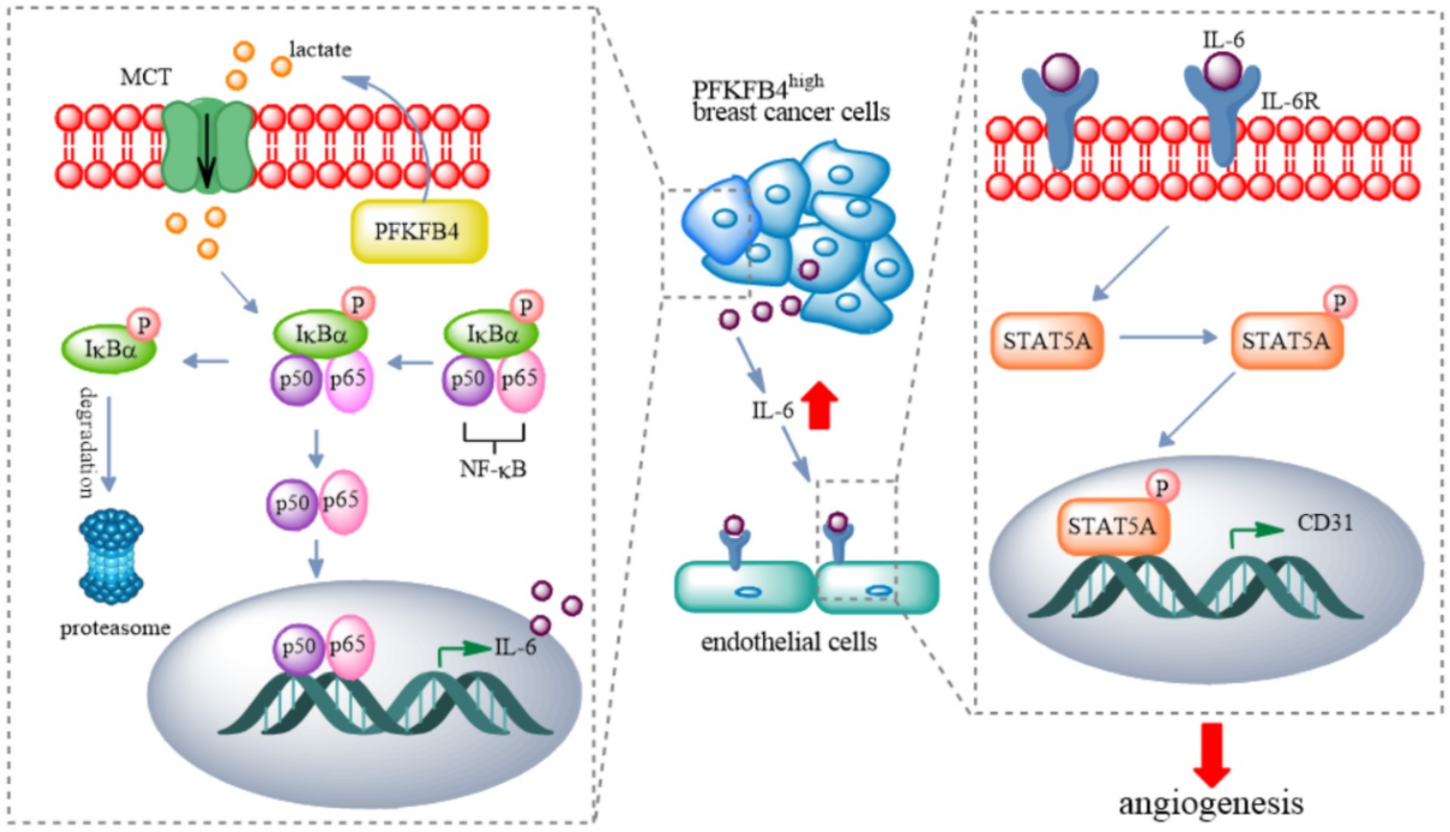
** A proposed model for PFKFB4-induced angiogenesis. PFKFB4 promotes angiogenesis via IL-6/STAT5A/P-STAT5 signaling in breast cancer.** PFKFB4 ectopic expression in breast cancer cells elevates lactate levels in the culture medium which initiates NF-κB activation and nuclear translocation. NF-κB within the nucleus binds to the IL-6 promoter region and then enhances IL-6 expression. The resultant IL-6 expression boosts IL-6R and CD31 (a vascular differentiation marker) expression in endothelial cells. Consequently, it appears that STAT5A/P-STAT5 (but not STAT3) is the pivotal signaling molecules involved in the angiogenic process.

In this study, we found that PFKFB4 promotes angiogenesis via IL-6/STAT5A/P-STAT5 signaling in breast cancer. PFKFB4 ectopic expression in breast cancer cells elevates lactate levels in the conditioned medium which initiates NF-κB activation and nuclear translocation. NF-κB within the nucleus binds to the IL-6 promoter region and then enhances IL-6 expression. The resultant IL-6 expression boosts IL-6R and CD31 (a vascular differentiation marker) expression in endothelial cells. Consequently, it appears that STAT5A/P-STAT5 (but not STAT3) are the pivotal signaling molecules involved in the angiogenic process (Fig. 7).

Previous reports have indicated that elevated PFKFB4 expression levels occur in various types of cancer (e.g., prostate cancer, bladder cancer, gastric cancer) 23-27. Elevated PFKFB4 expression levels have been associated with tumorigenesis 11, cell proliferation, metastasis 12, and drug resistance 13. Our study illustrates the novel finding that PFKFB4 promotes angiogenesis in breast cancer via IL-6 instead of vascular endothelial growth factor (VEGF) which is a well-established mediator of angiogenesis in cancer. Gopinathan et al. reported that defective pericyte coverage occurs in IL-6-stimulated vessel sprouts compared to VEGF-stimulated vessel sprouts using an aortic ring model although IL-6 induces endothelial cell proliferation and migration with similar potency to VEGF 28. However, Tzeng et al. reported that IL-6 induces VEGF expression and promotes angiogenesis via the apoptosis signal-regulating kinase 1 (ASK1)/p38/AP-1 pathway in human osteosarcoma 29. Huang et al. reported that IL-6 induces VEGF expression and angiogenesis via JAK/STAT pathway in gastric cancer 30. In addition, Wei et al. reported that IL-6 induces VEGF expression and angiogenesis via STAT3 pathway in cervical cancer 31. In contrast, our results clearly indicate that IL-6 does not induce VEGF expression in breast cancer. Our contrary results may be due to a number of factors which include the following: 1) the IL-6 concentration in breast cancer cells remains below the threshold required for inducing VEGF expression; 2) the time frame for IL-6-induced VEGF expression exceeds the time period we used in the study; and, 3) cell specific induction factors may exist in breast cancer cell that are not present in other cancers.

Our previous findings showed that PFKFB4 promotes lactate production and secretion in ZR-75-1 and SKBR3 breast cancer cells 17. In this study, we also showed that PFKFB4 promotes lactate production and secretion in MDA-MB-231 and T47D cells. Vegran et al. and Comito et al. have previously shown that lactate activates NF-κB signaling in endothelial cells and CD4+ T cells 32, 33. The activation of NF-κB signaling depends upon the cellular uptake of lactate which is facilitated by MCT. MCT1 and MCT3 are the main lactate transporters in PFKFB4-mediated NF-κB activation in our *in vitro* model. In this regard, we found that AZD3965 (a MCT1 inhibitor) treatment diminishes PFKFB4-induced NF-κB activation. It is well known that NF-κB regulates a large array of genes involved in multiple aspects of the immune and inflammatory responses. In regards to cancer, NF-κB participates in various facets of cancer initiation, development, metastasis, and resistance to treatment. Furthermore, various factors (e.g., TNFα, IL1β, EGF, LPS, ROS) are known to activate NF-κB and these factors may also regulate PFKFB4-mediated NF-κB activation 34. Therefore, we intend to explore the role of these factors in our future research.

STAT3 is a well-acknowledged downstream signaling molecule of IL-6. The IL-6/STAT3 signaling pathway consistently demonstrates aberrant hyper-activation in cancer patients. Therefore, inhibitors of IL-6/STAT3 signaling pathway are presently in clinical and/or preclinical development to inhibit tumor growth and relieve immunosuppression 35. However, we did not observe STAT3 activation with IL-6 stimulation in HUVEC endothelial cells. Instead, we detected STAT5 activation with IL-6 stimulation in HUVEC endothelial cells. Upon further investigation, we found STAT5A activation with IL-6 stimulation. Although STAT3 and STAT5 are structurally similar and activated by many of the same cytokine receptors, STAT3 and STAT5 demonstrate divergent or even opposing effects on gene expression and cellular phenotype. For example, VEGF increases STAT3 phosphorylation but not STAT5 phosphorylation that promotes endothelial cell migration and tube formation in human dermal microvascular endothelial cells 36. In addition, IL-3 activates STAT5 to induce pro-angiogenic signals in HUVEC 37. Tormo et al. found that STAT3 and STAT5 activation by IL-6 depends upon cytokine concentration and cytokine release time. STAT5 activation is transient and needs a higher concentration of IL-6 for activation than STAT3 does in CD4^+^ T cells 38.

In summary, we discovered that PFKFB4 plays a novel role in promoting angiogenesis in breast cancer. Specifically, we found that PFKFB4 promotes angiogenesis via IL-6 (not via VEGF) in breast cancer. Moreover, IL-6 exerts its angiogenic role via STAT5A/P-STAT5 in endothelial cells. 5-MPN (a specific PFKFB4 inhibitor) suppresses PFKFB4-induced angiogenesis. Consequently, we suggest that 5-MPN may serve either as a single agent or in combination with currently available treatments to improve the therapeutic outcome for breast cancer patients.

## Supplementary Material

Supplementary figure and tables.
