# PFKFB4 promotes angiogenesis via IL-6/STAT5A/P-STAT5 signaling in breast cancer: Erratum

**DOI:** 10.7150/jca.93775

**Published:** 2024-01-12

**Authors:** Dan Li, Jiaping Tang, Ruifang Gao, Jinxin Lan, Wenzhi Shen, Yanhua Liu, Yanan Chen, Hongwei Sun, Jie Yan, Yongwei Nie, Na Luo

**Affiliations:** 1 Department of Anatomy and Histology, School of Medicine, Nankai University, Tianjin 300071, China.; 2 Tianjin Institute of Medical & Pharmaceutical Sciences, Tianjin 300131, China.; 3 Department of Pathology and Institute of Precision Medicine, Jining Medical University, Jining 272067, China.; 4 Tianjin Key Laboratory of Tumour Microenvironment and Neurovascular Regulation, Nankai University, Tianjin 300071, China.; 5 Experimental Center of Operations, Chinese People's Armed Police Force Command College, Tianjin 300250, China.

In the original version of our article, there were 3 errors concerning misused images. Specifically,

- Fig 2D, the WB blots of IκBα and β-actin of T47D cells, β-actin of MDA-MB-231 cells and NF-κB of T47D cells are un-intentionally reused. We repeated the experiment and the correct image is provided below.
- Fig 5C, the WB blots of STAT5B of HUVEC cells treated with MDA-MB-231 CM or T47D CM are un-intentionally reused. We repeated the experiment and the correct image is provided below.
- Fig 6E, the IHC images of IL-6R of DMSO-MCS group and 5-MPN-PFKFB4 group are un-intentionally reused. The correct image is provided below.

The correction will not affect the results and conclusion. The authors apologize for any inconvenience this may have caused.

## Figures and Tables

**Fig 2D:**
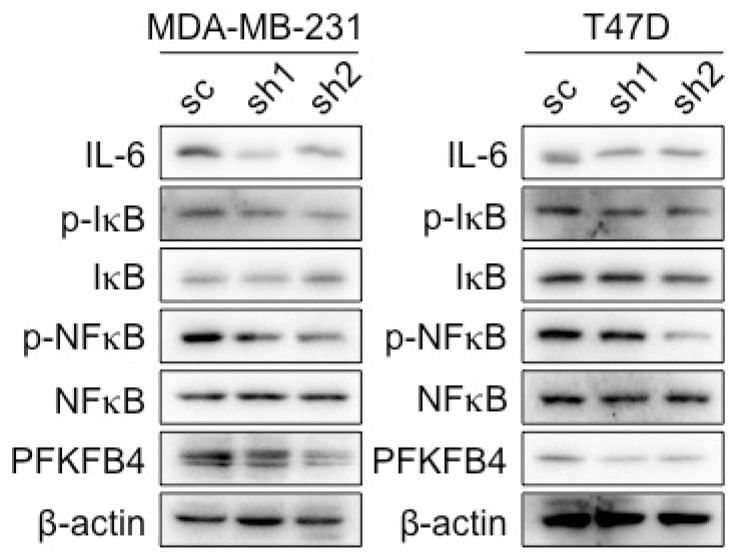
The Western blot analysis showed that knocking down of PFKFB4 in MDA-MB-231 and T47D cells decreased IκBα (S36) and NF-κB p65 phosphorylation. β-actin was used as a loading control.

**Fig 5C:**
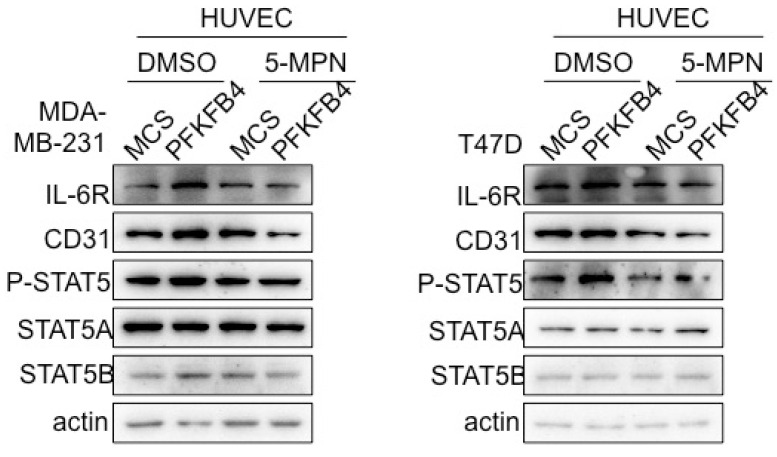
The Western blot analysis showed that 5-MPN treatment of MDA-MB-231 and T47D cells diminished PFKFB4-induced STAT5 phosphorylation and STAT5A, IL-6R, CD31 expression in HUVEC cells. β-actin was used as a loading control.

**Fig 6E:**
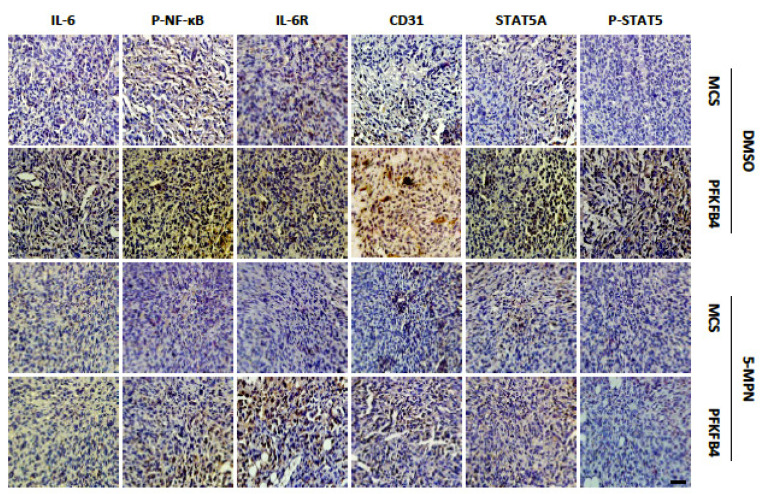
The representative micrographs of IL-6, P-NF-κB, IL-6R, CD31, STAT5A, and P-STAT5 immunocytochemical staining in xenograft MDA-MB-231 tumors. Ectopic expression of PFKFB4 increased immunocytochemical staining of above-mentioned molecules versus the MCS group, whereas, 5-MPN treatment inhibited PFKFB4-induced immunocytochemical staining of above-mentioned molecules. Scale bar= 50μm.

