# Supplementary material for: PFKFB4 promotes angiogenesis via IL-6/STAT5A/P-STAT5 signaling in breast cancer
Source: J Cancer. 2022 Jan 1;13(1):212–24. doi: 10.7150/jca.66773 (PMC8692682; doi:10.7150/jca.66773)
Supplement: Supplementary file 1 — Supplementary figure and tables. [file jcav13p0212s1.pdf]

Supplement Fig 1

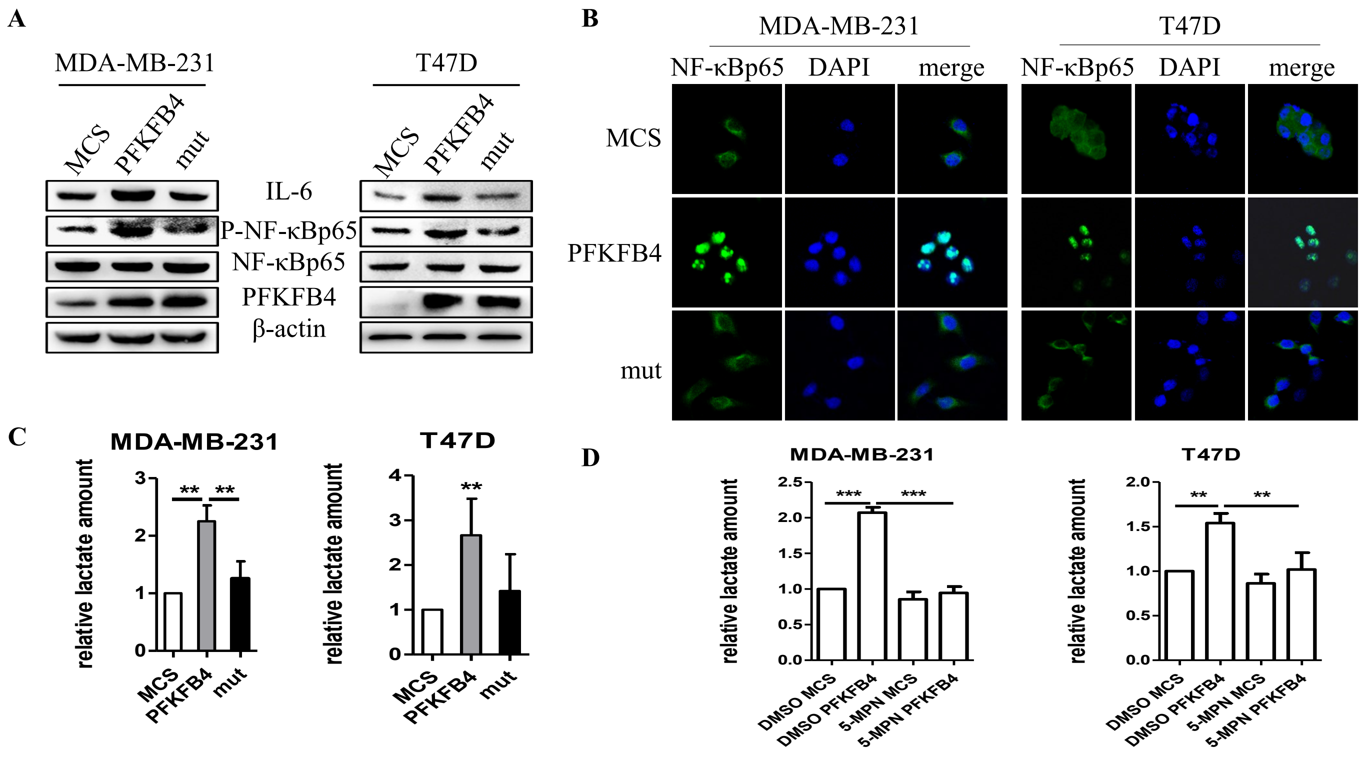

## Supplementary table 1. Primers for gene overexpression and shRNAs for gene knockdown

| Gene | Primer Sequences (5'→3') |
|---|---|
| PFKFB4 (Forward) | CGTCTAGAGCCACCATGGCGTCCCCACGGGAATTG |
| (Reverse) | CGACGCGTTCACTGGTGAGCAGGCACCGTG |
| PFKFB4-sh1 | AAAAGCCCAACTCTCATTGTCATGGTTGGATCCAACCATGACAATGAGAGTTGGGC |
| PFKFB4-sh2 | AAAAGCTGGAGAGGCAAGAGAATGTTTGGATCCAAACATTCTCTTGCCTCTCCAGC |

## Supplementary table 2. Primers for qRT-PCR

| Primer Name | Primer Sequence (5'->3') |
|---|---|
| **hIL6-F** | CTCAATATTAGAGTCTCAACCCCCA |
| **hIL6-R** | GAGAAGGCAACTGGACCGAA |
| **hVEGFA-F** | AGGGCAGAATCATCACGAAGT |
| **hVEGFA-R** | AGGGTCTCGATTGGATGGCA |
| **hPDGFA-F** | GCAAGACCAGGACGGTCATTT |
| **hPDGFA-R** | GGCACTTGACACTGCTCGT |
| **hbFGF-F** | CCACCTATAATTGGTCAAAGTGGT |
| **hbFGF-R** | TCATCAGTTACCAGCTCCCC |
| **hIL8-F** | TGGACCCCAAGGAAAACTGG |
| **hIL8-R** | TTGCTTGAAGTTTCACTGGCAT |
| **hMCT1 F** | GCGATCCGCGCATATAACG |
| **hMCT1 R** | GACAAAGTCTCCCAACCCCG |
| **hMCT3 F** | CCATGCTCTACGGGACAGG |
| **hMCT3 R** | GCTTGCTGAAGTAGCGGTT |

| | |
|---|---|
| **hMCT4 F** | CGTTTTTGTGCAGGTTTGGGT |
| **hMCT4 R** | CCCAGAACGGGCAATAGCTG |
| **hGAPDH F** | CTCTGATTTGGTCGTATTGGG |
| **hGAPDH R** | TGGAAGATGGTGATGGGATT |
